# Lactococcal 936-type phages and dairy fermentation problems: from detection to evolution and prevention

**DOI:** 10.3389/fmicb.2012.00335

**Published:** 2012-09-18

**Authors:** Jennifer Mahony, James Murphy, Douwe van Sinderen

**Affiliations:** ^1^Department of Microbiology, University College CorkCork, Ireland; ^2^Alimentary Pharmabiotic Centre, University College CorkCork, Ireland

**Keywords:** phage, lactic acid bacteria, *Lactococcus*, dairy, food fermentation

## Abstract

The so-called 936-type phages are the most frequently encountered lactococcal phage species in dairy fermentations, where they cause slow or even failed fermentations with concomitant economic losses. Several dairy phage population studies, performed in different geographical locations, have detailed their dominance in dairy phage populations, while various phage-resistance mechanisms have been assessed in a bid to protect against this virulent phage group. The impact of thermal and chemical treatments on 936 phages is an important aspect for dairy technologists and has been assessed in several studies, and has indicated that these phages have adapted to better resist such treatments. The abundance of 936 phage genome sequences has permitted a focused view on genomic content and regions of variation, and the role of such variable regions in the evolution of these phages. Here, we present an overview on detection and global prevalence of the 936 phages, together with their tolerance to industrial treatments and anti-phage strategies. Furthermore, we present a comprehensive review on the comparative genomic analyses of members of this fascinating phage species.

## INTRODUCTION

Phages infecting *Lactococcus lactis* are well-studied and represent a rather diverse group of bacterial viruses that are classified into 10 species based on morphology and sequence relatedness (Deveau et al., 2006). Eight of these ten species are Siphoviridae members, possessing long non-contractile tails, while the two remaining species belong to the Podoviridae family that are characterized by short tails (Deveau et al., 2006). Representatives of each of these species have been isolated through phage population studies of (predominantly) dairy facilities, although the most frequently isolated phages belong to the so-called 936, P335, and c2 species. The most frequently isolated phages in lactococcal dairy fermentations belong to the 936-type species and these phages therefore pose the highest threat to this industry. Furthermore, the virulent nature of these phages combined with their increased tolerance to sanitation and thermal treatments highlight the requirement for constant monitoring and analysis of this phage species.

## ISOLATION OF 936-TYPE PHAGES FROM GEOGRAPHICALLY DISTINCT LOCATIONS

The 936-type phage species were shown to clearly dominate lactococcal phage isolation studies as deduced from an extensive number of isolation studies across various geographical locations (**Table 1**).

**Table 1:** Isolation of dairy phages from different geographical locations

Region	Phage species isolated	Source	Starter type (if known)	Reference
Argentina	936 and P335	Milk	Commercial, defined	Suarez et al. (2008)
Canada	936	Cheese whey	Defined	Rousseau and Moineau (2009b)
Denmark	936	Cheese whey	Mixed	Josephsen et al. (1999)
Ireland	936 and P335 (prophage)	Cheese whey	Mixed	Casey et al. (1993); Arendt et al. (1994)
Norway	936 and P335 (prophages)	Cheese whey/Bulk	Commercial, defined	Kleppena et al. (2011)
		starter material		
Poland	936 and c2	Cheese whey	Commercial	Szczepanska et al. (2007)
Republic of Belarus	936 and c2	Milk	Commercial	Raiski and Belyasova (2009)
Slovenia	936 and c2	Cheese whey	Commercial	Miklič and Rogelj (2003)
United States	936, c2, and P335	Buttermilk	Commercial	Moineau et al. (1996)
Australia	936	Cheese whey	Commercial	Castro-Nallar et al. (2012)

In 1993, 22 phages of the 936-type species with its classic morphology of a long non-contractile tail and isometric protein head were isolated from cheese whey (Casey et al., 1993). Similarly, over an 11 month period, twenty-two 936-type phages were isolated from a buttermilk fermentation facility, accounting for 80% of the total phage species isolated (Moineau et al., 1996). Interestingly, the latter study identified raw milk as the major source of phage contamination as buttermilk fermentation occurs in closed vats. It was also observed that 936-type phage isolates from distinct geographical regions had significant similarities in terms of host range and phage restriction profiles. In 1992, 30 lactococcal phages were isolated from Canadian (province of Quebec) cheese factories, of which eleven were found to belong to the 936-type species as determined by electron microscopy, DNA homology, and protein profiles (Moineau et al., 1992). Interestingly, phages belonging to the c2-type species were found to be the most frequently isolated in this particular study. In a more recent survey, phages belonging to the 936-type species have emerged to dominate phage populations in Canadian whey samples. Out of 71 lactococcal phages isolated against a mixed starter culture, the 936 species accounted for 74% and were found to exhibit a wide host spectrum (Bissonnette et al., 2000).

In stark contrast to the Canadian, Irish, and U.S. studies, analysis of phage prevalence of six Belarusian dairy regions showed that just four out of 23 isolated lactococcal phages were shown to belong to the 936-type species (Raiski and Belyasova, 2009). The 936-type phages BIM BV-27, E11, and E12 all had similar restriction profiles yet were isolated from central, western, and south-western regions in Belarus, further emphasizing the widespread proliferation of this phage species with limited changes in their genetic content. This is not precluding the possibility that these fermentation plants obtained their starter cultures from the same supplier(s), thus representing a limited number of strains with a consequently restricted level of phage diversity. Ecological analysis of phage biodiversity in several regions of Belarus’ neighboring country, Poland, identified members of the 936-type species as the dominant phage found in Polish dairy plants (Szczepanska et al., 2007). Two particular isolates of the 936-type species were found in more than one location, bIBB5g_1_ and bIBBEg_1_, which were isolated from three and seven of the 17 tested locations, respectively. Further affirming the dominance of the 936-type phages in a global context, members of this species were the shown to be the most frequently isolated in Norwegian and U.S. whey samples originating from Dutch type cheese fermentations (Kleppena et al., 2011). In total, twenty-five 936-type phages were detected in the latter study, which were shown to be distinct based on the sequence of a DNA fragment encoding structural phage proteins. This section of the genome contains a unique region that was identified as a mutation hotspot which defined the individual isolates, highlighting the diversification of these phages.

Phages have also been isolated from various locations within the dairy facility other than the dairy product production line. Several studies have demonstrated that phages have the ability to become airborne within a dairy facility, more than likely due the use of open vats, processing conditions, and movement of equipment (Neve et al., 1994, 1995, 2003; Verreault et al., 2011). In the Verreault et al. (2011) study, aerosol samples were taken at the filling section at the end of the production line within a cheese factory, where 936-type phages were detected in the air at levels of 10^4^ phage genomes/m^3^, a level significant enough to affect cheese production if access to open vats was to be gained. Furthermore, phages were isolated from surfaces such as doors, cleaning materials, offices, and floors, with the highest concentration of 936-type phages at 9106 ± 789 genomes cm^-^^2^. Such contamination levels create an environment whereby staff could spread phages across the plant, making efforts to control such populations very difficult. Not only are the 936-type phages prominent within the lactococcal dairy industry as evident from biodiversity studies, this particular species appears to possess an intrinsic ability to increase its virulence. Josephsen et al. (1999) compared 936-type phages isolated between 1989 and 1994, and 936-type isolates from 1982 to 1986, showing that both phage sets shared homology to each other but that the more recent isolates exhibited a broader host range and a greater burst size, thus indicating that the phages found in this particular plant had evolved to become more infective. The 936-type phages have also been shown to persist in a specific dairy factory during an extended period of time, as demonstrated by the isolation and subsequent re-isolation of the 936-type phage CB13/GR7 (Rousseau and Moineau, 2009a). Moreover, this study suggested that newly isolated phages were derived in some part from the older isolates, highlighting that the 936 phage species continues to retain dominance due to its genetic elasticity and ability to evolve to a changing environment.

## DETECTION OF 936-TYPE PHAGES

Two main methods for the detection and enumeration of phages from industrial dairy plants are used. The first method, which is predominantly used by the dairy industry for phage monitoring purposes, is the so-called Heap–Lawrence test, which is based on the acidification rate of milk and provides a reliable test for the presence of phages, while it also gives an indication of phage titer (Heap and Lawrence, 1976). The second one, used most intensely by research laboratories is the standard double-layer plaque assay (Lillehaug, 1997), which following overnight incubation of the plates allows for the quantitative analysis of phage levels. In recent years, a number of alternative molecular methods have been developed to identify phages present in dairy samples. One such method is the use of polymerase chain reaction (PCR), which allows the reliable detection and distinction of the three most common phage species (936, c2, and P335) in a single multiplex primer-based PCR reaction (Labrie and Moineau, 2000). In terms of the 936-type phage, the major structural protein was shown to be highly conserved (approximately 86% identity at DNA level) among the phages examined (p2, Q7, and Q11 sk1, bIL170, and F4-1; Labrie and Moineau, 2000). Primers were designed to target this gene, generating an amplicon of 179 base pairs (bp) and allowing a detection limit of 10^4^ pfu/ml (plaque forming units per ml of sample). A similar method was developed (del Rio et al., 2007) based on the *msp* gene of phages p2 and Q7, the *orf11* gene of phage sk1, the *orf13* gene of phage bIL170, and the *mcp* gene of phage F4-1, all belonging to the 936-type species and lowering the detection limit to 10^3^ pfu/ml. While both PCR methods are effective at detecting and discriminating phages in raw milk and whey, they do not permit the isolation of individual phages. The receptor binding protein (RBP) of the 936-type phage is one of the more divergent regions of the genome and is believed to be responsible for the phage’s host range (Dupont et al., 2004). Using this information, Dupont et al. (2005) developed an optimized magnetic capture hybridization (MCH) PCR-based assay to detect 936-type phage and to further detect the different subgroups of this species with primers targeting the variable region of the RBP gene at a detection limit of 10^2^–10^3^ pfu/ml (Dupont et al., 2005). However, recent studies have highlighted that emerging 936-type phages no longer fall into these originally identified RBP-based subgroups, thus advocating primer redesign for future multiplex-based phage detection and subgroup assignment (Castro-Nallar et al., 2012). Real-time (RT) PCR, another molecular technique that allows the rapid detection and quantification of microorganisms has also been used for the detection 936-type phages (Ly-Chatain et al., 2011; Verreault et al., 2011). As mentioned above, the Verreault et al. (2011) study reported the detection of airborne 936-type phages within a dairy environment using RT-PCR with a detection limit of 10^2^–10^4^ pfu/ml. The Ly-Chatain et al. (2011) study applied RT-PCR to DNA isolated from milk and whey, and achieved a detection limit of 10^2^ pfu/ml. Both studies would permit rapid sampling and identification of phages in given industrial plant to gage phage contamination levels, however, they do not permit the isolation of individual isolates within a given species and also would not detect the emergence of rare or new phages. One method to overcome such limitations employs the use of flow cytometry (Michelsen et al., 2007). Phage infection of a host cell can be monitored in real time by measuring loss of host cell mass using light scatter and DNA fluorescence staining, allowing a detection limit of 10^4^–10^5^ pfu/ml. It is clear from the numerous biodiversity studies on lactococcal phages that a large quantity of diverse 936-type phages can be isolated from any given factory. Host range analyses and restriction profiling of phage genomes is time consuming and may require numerous attempts to achieve suitable patterns for comparative analysis between phages. A multilocus sequence typing (MLST) scheme was developed using primers that target the middle region of conserved genes found in 936-type phages (Moisan and Moineau, 2012). This approach utilizes a single PCR reaction using five primers sets targeting the genes encoding the major capsid protein (MCP), tape measure protein (TMP), lysin (lys), large terminase (terL), and the major tail protein (MTP). Of note, the PCR can be performed on phage lysates, eliminating the need to isolate phage DNA.

A number of methods are now available to both industry and academics alike to characterize the biodiversity of phages in a dairy factory, and to enumerate and detect phages that belong to the 936-type species. For industrial purposes, rapid detection of phages by PCR-based methods may be more applicable so a timely intervention can be introduced to prevent fermentation failure. For research, it is necessary to isolate individual 936-type phages present within a specific environment to allow continued study into why these phages are so prevalent and how they overcome technological hurdles. The standard plaque assay followed by molecular characterization may remain the best methodologies to perform such studies.

## PHYSICAL APPROACHES TOWARD INACTIVATION OF 936-TYPE PHAGES

In order to prevent the negative consequences of phage infection in the dairy factory, i.e., the development of slow or dead vats, various counter measures are employed, such as thermal and biocidal treatments, strain rotation regimes and the application of bacteriophage-insensitive strains (Lavigne et al., 2009; Mills et al., 2010). A number of reports have been published on the thermal and biocidal elimination of phages for lactobacilli, streptococcal, and lactococcal phages (Quiberoni et al., 2003; Muller-Merbach et al., 2005a; Atamer and Hinrichs, 2009), which have indicated that the level of resistance/sensitivity varies between phage species, and that certain phages appear to be quite resilient to such treatments (Avsaroglu et al., 2007).

### THERMAL INACTIVATION

In the dairy industry a number of temperature treatments are applied to the incoming raw milk depending on the product. These include both low temperature long time (63°C for 30 min) and high temperature short time (72°C for 15 s) for Cheddar cheese, while either a treatment of 80°C for 30 min or 95°C for 10 min is used for yogurt production (Soukoulis et al., 2007). These temperature treatments are applied to achieve adequate pasteurization of the raw milk, however, most of these are less harsh than the temperature exposure of 90°C for 15 min to inactivate phage recommended by the International Dairy Federation to achieve complete phage inactivation (Svensson and Christiansso, 1991).

Raw milk is a constant source of phages (McIntyre et al., 1991) that threaten fermentation processes in a dairy plant due to the ability of a certain percentage of the phage population to remain viable following pasteurization, as was confirmed by Madera et al. (2004). This study further demonstrated that the 936-type phages were significantly more resistant to heat treatments as compared to the other two predominant lactococcal phages (i.e., those that belong to the P335 and c2-type species). The ability of 936-type phage to withstand pasteurization was also observed for phages P001 and P008, with active phage particles still detectable following a 10 min and 60 min exposure, respectively (Muller-Merbach et al., 2005b). The assumption of raw milk being an important source for phage contamination was further supported by the ability of phage P008 to remain detectable at 80°C (Muller-Merbach et al., 2005a). Additionally, this study demonstrated that milk in fact protects against thermal inactivation of phages, an observation corroborated by several other studies (Quiberoni et al., 2003; Atamer et al., 2010).

In addition to surviving pasteurization, several lactococcal 936-type phages have emerged with remarkably higher thermal resistance. Atamer and Hinrichs (2009) reported the isolation of twenty-two 936-type phages that exhibited the ability to survive a temperature exposure of 80°C for 5 min. P335- and c2-type phages were also isolated in this study, however, only one of the P335-type phages and none of the c2-type phages were shown to be resistant to 80°C. Active phage particles for four of the 936-type phages, i.e., P680, P656, P1532, and P4565, were also observed after a 5 min exposure to 85 and 90°C. Further characterization of P680 showed that a 6-log reduction in infectivity was achieved following exposure to 90°C for 15 min (Atamer and Hinrichs, 2010). These studies highlight the emergence of highly thermo-tolerant phage within the 936-type phage species, and, along with the protective nature of milk, it may account for the dominance of this particular species in dairy industries globally.

### BIOCIDAL INACTIVATION

A range of strategies are utilized within a dairy setting to control phage populations, one such approach being the use of biocidal agents. These include sodium hypochlorite, peracetic acid, and ethanol, and the impact of these agents on bacteriophage infectivity has been recently reviewed (Guglielmotti et al., 2011). Despite extensive characterization of the biocidal inactivation of phage infecting lactic acid bacteria (LAB) species other than *L. lactis *(Quiberoni et al., 1999; Quiberoni et al., 2003; Capra et al., 2004), only a limited number of investigations have been carried out to study inactivation of 936-type phages using such antimicrobial agents. One study showed that the 936-type phage QF12 was completely inactivated at a concentration of 200 parts per million (ppm) sodium hypochlorite for 45 min and a T_99_ value (the time required to inactivate 99% of phage infectivity) of 1.9 min and 3.8 min in 100% ethanol and isopropanol, respectively (Suarez and Reinheimer, 2002). Peracetic acid, a biocidal agent with a low pH, is known to be effective in reducing infectivity of bacteriophages, including those belonging to the 936-type species (Suarez and Reinheimer, 2002). Other investigations into the effectiveness of biocidal inactivation of lactococcal phages have reported that the impact of such chemicals can be biocide- and phage-dependent, however, the species identity of the tested phages was not stated (Parada and Fabrizio, 2001; Avsaroglu et al., 2007; Buzrula et al., 2007).

A more recently described approach with limited research carried out to date is the use of TiO_2_ to induce a photolytic reaction generating highly oxidizing species when exposed to ultra-violet (UV) light (Briggiler Marcóa et al., 2011). Two 936-type phages, CHD and QF9, were shown to be completely eliminated within 120 and 60 min of exposure, respectively. While this would be considered a rather lengthy procedure the study also suggested that levels required have no associated negative impact on human health. Therefore, and with the right design, equipment can be incorporated in a factory setting to constantly expose the processing plant to photocatalytic activity, so as to reduce the overall number phages while also minimizing the spread of phages between surfaces, by staff and through the air.

## 936 PHAGES RESPONSE TO PHAGE-RESISTANCE SYSTEMS

In addition to physical and chemical barriers that phages encounter in fermentation facilities, starter cultures themselves present many biological hurdles to phage infection in the form of phage-resistance systems. During the past three decades, a very significant volume of data relating to the response of various lactococcal phages to phage-resistance systems has emerged. Many of these studies also highlight that while significant levels of protection against these phages are afforded by these systems, phage variants may arise that can bypass these systems and therefore have the potential to persist in dairy fermentation facilities (Gabs and Josephsen, 2003; Fortier et al., 2005; Haaber et al., 2008). Abortive infection (Abi) systems and restriction/modification (R/M) systems are widespread in the genomes and plasmids of lactococcal strains, and have been studied extensively with a view to improving dairy starter cultures (Gabs and Josephsen, 2003; O’Driscoll et al., 2004; Chopin et al., 2005). These systems dominated studies of lactococcal phage-resistance in the 1990s and the first decade of the current century (Garvey et al., 1995; Daly et al., 1996), and consequently there is a wealth of information relating to the response of 936-type phages to such resistance systems. For example, all Abi systems identified to date provide resistance against 936-type phages with the exception of AbiR and AbiZ, for which the effects have not been well defined (Twomey et al., 2000; Chopin et al., 2005; Durmaz and Klaenhammer, 2007). Several studies of R/M systems also detail the sensitivity of 936-type phages to such systems (Twomey et al., 2000; Boucher et al., 2001; Kong and Josephsen, 2002; O’Driscoll et al., 2004). Similarly, superinfection exclusion (Sie) systems encoded by the genomes of lactococcal prophages have been tested against a variety of 936 phages and are effective against members of this phage species (McGrath et al., 2002; Mahony et al., 2008). Interestingly, a prophage-encoded Sie system from a *Streptococcus thermophilus*-infecting phage is also effective against the 936-type phages, when applied in a lactococcal host background (Sun et al., 2006). This unexpected (as *S. thermophilus* is not known to be sensitive to 936-type phages) finding suggests that this gene was acquired by horizontal transfer from a lactococcal starter and may be reminiscent of the complex and symbiotic evolutionary relationships between these starter bacteria.

While the 936-type phages appear to be sensitive to the majority of lactococcal anti-phage strategies, it is apparent that variant phages, which have overcome the imposed resistance system, are readily isolated in many cases. Possibly the best described example of this is the mutation of the *sak* gene of the 936 phage, p2, to overcome the Abi system AbiK (Boucher et al., 2000; Bouchard and Moineau, 2004). Similarly, genes that are involved in sensitivity to the abortive infection AbiD1 have been identified in another 936-type phage, bIL66 (Bidnenko et al., 1995). The deduced product of one of these genes, *Orf3*, displays similarity to a putative Holliday junction endonuclease of lactococcal phages and has homologs on the genomes of many 936 phages at the rightward end of the genome within the so-called “middle-expressed” module. The relative ease with which such mutations are acquired highlights the powerful adaptive responses of these phages to biological hurdles.

## 936 PHAGE GENOMICS: EVOLUTIONARY ASPECTS

To date, complete genomes of forty-one 936-type phages have been sequenced and are publicly available (Chandry et al., 1997; Crutz-Le Coq et al., 2002; Mahony et al., 2006; Hejnowicz et al., 2009; Rousseau and Moineau, 2009a; Castro-Nallar et al., 2012). The first sequence to be published was that of sk1 and in the 15 years ensuing this, a wealth of sequence data has become available permitting extensive comparative analyses within this species (Chandry et al., 1997). The genome sequence of bIL170 provided molecular proof for early suggestions of a bipartite sub-classification based on the phage’s ability to infect either *L. lactis *ssp. *lactis *or *L. lactis *ssp. *cremoris *(Crutz-Le Coq et al., 2002; Dupont et al., 2004). Several comparative genomic analyses have since reported that there are a number of genomic regions of divergence, most particularly within their presumed replication module (Mahony et al., 2006; Rousseau and Moineau, 2009a; Castro-Nallar et al., 2012).

**FIGURE 1:**
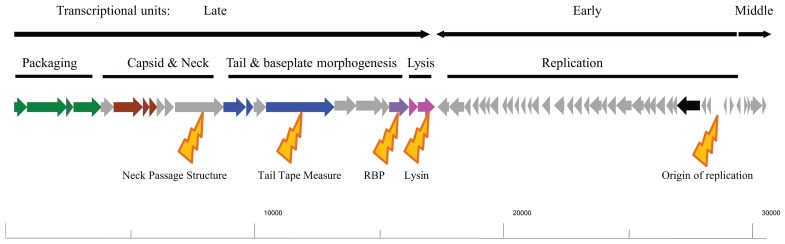
**Schematic representation of the typical genome architecture of a typical 936 phage.** Genes with a predicted or proven function are assigned colors: green arrows indicate genes encoding the terminases and portal protein involved in phage DNA packaging; red arrows indicate the capsid genes; Blue arrows indicate tail structural genes; the purple arrow represents the receptor binding-encoding gene; pink arrows represent the holin and lysin of the lysis module; the black arrow represents a (subunit of) DNA polymerase. The transcriptional units as shown for sk1 are highlighted above the genomic drawing as are the functional modules of the genome. Below the genomic drawing are yellow highlighters of the dominant points of divergence in the genomes of the 936 phages: the neck passage structure, the tail tape measure, the receptor binding protein, lysin, and the origin of replication, notwithstanding that a general divergence is observed in the replication module in general. The indicated scale at the bottom of the figure represents base numbering starting at the *cos* site.

The genomes of the 936-type phages are generally between 26 and 32 kb in length, and contain up to 65 open reading frames organized into three transcriptional units: the first in a rightward orientation encoding the morphogenesis module, which is head-to-head with the second transcriptional unit. The latter is involved in DNA replication and is followed by the third unit, for which a function is not established yet and which is in a back-to-back orientation with the replication module (**Figure 1**; Chandry et al., 1997). These three transcriptional units are conserved in all sequenced 936 phages with point mutations and insertions/deletions being the main source of divergence between such phages.

As mentioned above, the early expressed replication module is where the majority of divergence is observed between the sequenced phages, however, many of the predicted genes have no functional assignments with the exception of a putative DNA polymerase subunit and a single-stranded binding protein (Mahony et al., 2006; Rousseau and Moineau, 2009a). This makes the replication module essentially a black box, although some elements within this module have been subjected to detailed analysis such as the *sak *gene of p2 (Boucher et al., 2000; Bouchard and Moineau, 2004). This is in stark contrast to the well-studied P335 phages, for which many of the replication genes have been assigned functions (McGrath et al., 1999; Labrie et al., 2008).

The most intensely characterized of the three functional modules is the morphogenesis module and predicted functions have been assigned to most of the genes in this operon either through sequence relatedness to previously characterized phages or functional assessments. Within the structural module (the late-expressed transcriptional unit) genes involved in packaging (large and small terminase subunits), the capsid, portal protein, neck passage structure, tail tape measure protein and its associated chaperone, tail structural genes and the tail tip (RBP-encoding gene) and genes involved in lysis have been identified (**Figure 1**; Dupont et al., 2004; Mahony et al., 2006; Siponen et al., 2009a). It is the divergence of individual components of this module that has formed the basis for much of the more recent in-depth sub-classification of the 936 phages (Mahony et al., 2006; Rousseau and Moineau, 2009a; Castro-Nallar et al., 2012). Of the above-mentioned gene products, the presence of a so-called “neck passage structure” and the divergence of genes encoding the tail tape measure, the RBP and the lysin have been implicated as the major points of diversification within the structural module of these phages (**Figure 1**; Mahony et al., 2006).

The neck passage structure is encoded by gene *l12* of bIL170, which is located between the genes specifying the head and tail structural elements, and has representative homologs on many of the sequenced 936 phages (Crutz-Le Coq et al., 2002). It bears similarity to a host recognition domain identified in the temperate P335-type phages bIL309 and BK5-T. It is a structural element that is not required for propagation or tail assembly but is proposed to be involved in host range extension or determination (Crutz-Le Coq et al., 2006). This gene, or clear homologs thereof, has been identified in the genomes of bIL170, P008, 712, bIBB29, CB13, CB14, CB19, and CB20, as well as in 16 out of 28 phages isolated and sequenced in a recently published phylogeographical study (Mahony et al., 2006; Rousseau and Moineau, 2009a; Castro-Nallar et al., 2012). While this gene product is not essential, it has been suggested that its presence may provide a competitive advantage to phages possessing it (Crutz-Le Coq et al., 2006; Mahony et al., 2006). The presence and evolution of such genes provide one of the primary indicators of the environmental pressure imposed on these phages to adapt to their surroundings; the bacterial hosts available to the phages, the seemingly inexhaustible number of host-encoded phage-resistance mechanisms and harsh thermal and chemical treatments which phages have to cope with in modern dairy facilities. The presence or absence of this gene was one of the first points upon which phylogenetic sub-grouping of the 936 phages was based as those phages that were known at that time to infect *L. lactis *ssp. *lactis* strains possessed a homolog of this gene (bIL170, P008), while those known to predominantly infect *L. lactis *ssp. *cremoris *strains did not (jj50, sk1; Mahony et al., 2006). Additionally, it was proposed that there may a further sub-group that is intermediate in terms of host range and these (represented by 712) possessed a truncated derivative of the gene relative to bIL170 (Mahony et al., 2006).

**FIGURE 2:**
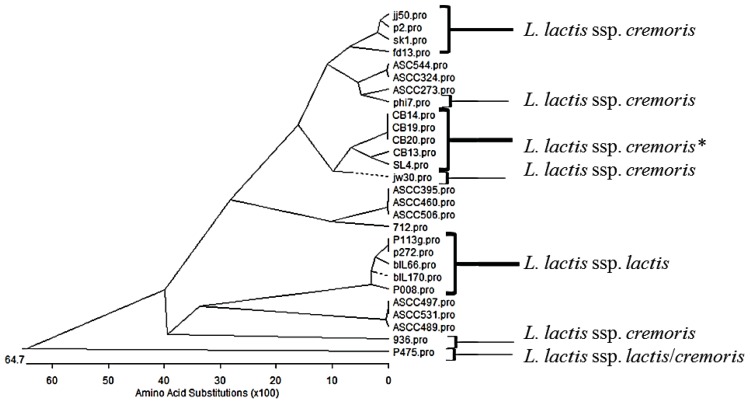
**Phylogenetic tree of the receptor binding proteins (RBP) of representatives of the 936 phage species from several studies** (Dupont et al., 2005; Mahony et al., 2006; Rousseau and Moineau, 2009a; Castro-Nallar et al., 2012). Phages that have been demonstrated to infect specifically *L. lactis *ssp. *cremoris *or ssp. *lactis*, are highlighted and P475 which infects both subspecies is also highlighted. *Indicates phages that have been propagated on a *L. lactis *ssp. *cremoris *strain but whose host range data is unknown. The remainder (ASCC collection) have a known host range but this data is unpublished. This phylogenetic tree highlights the divergent nature of the RBP sequences and while many of the sub-groups at the top of the tree are *L. lactis *ssp. *cremoris*-infecting phages and the lower half of the tree may be dominated by *lactis*-infecting phages, there are many of unknown host range.

More significantly, there has been a tremendous surge in data relating to the identification, structural analysis and phylogenetics of RBPs of the 936-type phages (Dupont et al., 2004; Ricagno et al., 2006; Spinelli et al., 2006; Tremblay et al., 2006; Sciara et al., 2010; Castro-Nallar et al., 2012). This increased interest in the phylogeny of the 936-type RBPs has permitted a more in-depth analysis of the relationship between host range and the phylogeny of the RBPs. While it was initially suggested (Mahony et al., 2006) that there may be a tripartite subgrouping of the phylogeny of the 936-type phages based on sequence divergence of particular genomic regions, including the gene encoding the RBP, it now appears that this may be much more complex as additional genome sequences become available (**Figure 2**; Mahony et al., 2006). The RBP sequences are conserved at the N-terminus of the encoded proteins, while the C-terminus is variable reflecting the varied interactions that the phages has with carbohydrates on the cell surface of the lactococcal host (Siponen et al., 2009b). While there are many 936-type phages that seemingly infect either *L. lactis *ssp. *lactis *or *cremoris *strains, it is also apparent that many of the phages within this species are capable of crossing the subspecies barrier and many more whose host range is as yet undefined but may potentially be predicted based on the RBP phylogeny (**Figure 2**). This may implicate two scenarios relating to the RBP sequence: (1) the RBP C-terminus is capable of interacting with multiple carbohydrates or (2) the RBP C-terminus is interacting with a carbohydrate element which is not specific to a particular subspecies. The latter scenario seems the more plausible although this is currently conjecture. It is also noteworthy that while there is significant interest in the phylogeny of the RBPs of these phages, it is of little benefit to understanding the relationship between host range and RBP sequence without information on the host range of the phages. For example, the RBP phylogenetic tree presented here (**Figure 2**) highlights sub-groups capable of infecting either *L. lactis *ssp. *lactis *or *cremoris*. These sub-groups are interjected with sub-groups for which host range data is unknown or poorly defined (**Figure 2**). Predictions on host range could be predicted based on the phylogeny of the RBPs but this is merely speculation until biological data is available.

Sequence comparisons of the tail tape measure, lysine, neck passage structure, and RBP proteins encoded by the various 936-type phages have shown that they seem to follow parallel evolutionary pathways in that observed divergence in one of these proteins in a given phage is normally accompanied by a similar level of divergence of any of the other mentioned proteins in that same phage (Mahony et al., 2006). A similar observation is made when examining the genomic termini (cohesive ends) and the origin of replication of the phage at the rightward end of the genome (Mahony et al., 2006). This indicates a common ancestral pathway for the 936 phages with diversification in certain elements to provide new derivatives with increased competitiveness. As more 936 genome sequences become available, a more confident phylogeny of these phages will be established and thus continued monitoring of dairy plants and environmental samples will define newer populations and will provide information on how the species is adapting to overcome new anti-phage strategies so as to infect starter strain mixes in the dairy setting.

## FUTURE PERSPECTIVES

The 936-type phages continue to dominate as the single most problematic lactococcal dairy phage species. Although significant data exists relating to the 936 phages, it is evident through genomic and evolutionary studies and furthermore through physical characterization studies that this species is continually evolving to overcome biological and physical hurdles pitted against them. This evokes a warning that constant monitoring of this species at the genomic level is of great significance in order to understand the phage population dynamics within any plant. The constant evolution of newer isolates with increasing resistance to thermal, chemical, and biological barriers requires rapid responses from the dairy industry by developing resistant starter strains and identifying alternative phage-resistant starter strains. This fascinating species of lactococcal phages is an excellent model to study the evolutionary processes within a closely related species of phages and highlights the relative importance of these phages in the dairy industry but also to the broader study of phage biology and evolution.

## Conflict of Interest Statement

The authors declare that the research was conducted in the absence of any commercial or financial relationships that could be construed as a potential conflict of interest.

## References

[B1] Arendt E. K. Daly C. Fitzgerald G. F Van De Guchte M. (1994). Molecular characterization of lactococcal bacteriophage Tuc2009 and identification and analysis of genes encoding lysin, a putative holin, and two structural proteins. *Appl. Environ. Microbiol.* 60 1875–1883 8031083 10.1128/aem.60.6.1875-1883.1994 PMC201575

[B2] Atamer Z. Hinrichs J. (2009). Thermal inactivation of the heat-resistant *Lactococcus lactis* bacteriophage P680 in modern cheese processing. *Int. Dairy J.* 20 163–168

[B3] Atamer Z. Dietrichb J. Neveb H. Hellerb K. J. Hinrichsa J. (2010). Influence of the suspension media on the thermal treatment of mesophilic lactococcal bacteriophages. *Int. Dairy J.* 20 408–414

[B4] Atamer Z. Hinrichs J. (2010). Thermal inactivation of the heat-resistant *Lactococcus lactis* bacteriophage P680 in modern cheese processing. *Int. Dairy J.* 20 163–168

[B5] Avsaroglu M. D. Buzrula S. Alpasa H. Mustafa A. (2007). Hypochlorite inactivation kinetics of lactococcal bacteriophages. *LWT Food Sci. Technol.* 40 1369–1375

[B6] Bidnenko E. Ehrlich D. Chopin M. C. (1995). Phage operon involved in sensitivity to the *Lactococcus lactis* abortive infection mechanism AbiD1. *J. Bacteriol.* 177 3824–3829 7601849 10.1128/jb.177.13.3824-3829.1995 PMC177102

[B7] Bissonnette F. Labrie S. Deveau H. Lamoureux M. Moineau S. (2000). Characterization of mesophilic mixed starter cultures used for the manufacture of aged cheddar cheese. *J. Dairy Sci.* 83 620–627 10791775 10.3168/jds.S0022-0302(00)74921-6

[B8] Bouchard J. D. Moineau S. (2004). Lactococcal phage genes involved in sensitivity to AbiK and their relation to single-strand annealing proteins. *J. Bacteriol.* 186 3649–3652 15150253 10.1128/JB.186.11.3649-3652.2004 PMC415755

[B9] Boucher I. Emond E. Dion E. Montpetit D. Moineau S. (2000). Microbiological and molecular impacts of AbiK on the lytic cycle of *Lactococcus lactis* phages of the 936 and P335 species. *Microbiology* 146(Pt. 2) 445–453 10708383 10.1099/00221287-146-2-445

[B10] Boucher I. Emond E. Parrot M. Moineau S. (2001). DNA sequence analysis of three *Lactococcus lactis* plasmids encoding phage resistance mechanisms. *J. Dairy Sci.* 84 1610–1620 11467810 10.3168/jds.S0022-0302(01)74595-X

[B11] Briggiler Marcóa M. del Luján Quiberonia A. Negrob A. C. Reinheimera J. A. Alfanob O. M. (2011). Evaluation of the photocatalytic inactivation efficiency of dairy bacteriophages. *Chem. Eng. J.* 172

[B12] Buzrula S. Öztürkb P. Alpasa H. Akcelik M. (2007). Thermal and chemical inactivation of lactococcal bacteriophages. *LWT Food Sci. Technol.* 40 1671–1677

[B13] Capra M. L. Quiberoni A. Reinheimer J. A. (2004). Thermal and chemical resistance of *Lactobacillus casei* and *Lactobacillus paracasei* bacteriophages. *Lett. Appl. Microbiol.* 38 499–504 15130146 10.1111/j.1472-765X.2004.01525.x

[B14] Casey C. N. Morgan E. Daly C. Fitzgerald G. F. (1993). Characterization and classification of virulent lactococcal bacteriophages isolated from a Cheddar cheese plant. *J. Appl. Microbiol.* 74 268–275

[B15] Castro-Nallar E. Chen H. Gladman S. Moore S. C. Seemann T. Powell I. B. Hillier A. Crandall K. A. Chandry P. S. (2012). Population genomics and phylogeography of an Australian dairy factory derived lytic bacteriophage. *Genome Biol. Evol.* 4 382–393 22355195 10.1093/gbe/evs017 PMC3318435

[B16] Chandry P. S. Moore S. C. Boyce J. D. Davidson B. E. Hillier A. J. (1997). Analysis of the DNA sequence, gene expression, origin of replication and modular structure of the *Lactococcus lactis* lytic bacteriophage sk1. *Mol. Microbiol.* 26 49–64 9383189 10.1046/j.1365-2958.1997.5491926.x

[B17] Chopin M. C. Chopin A. Bidnenko E. (2005). Phage abortive infection in lactococci: variations on a theme. *Curr. Opin. Microbiol.* 8 473–479 15979388 10.1016/j.mib.2005.06.006

[B18] Crutz-Le Coq A. M. Cantele F. Lanzavecchia S. Marco S. (2006). Insights into structural proteins of 936-type virulent lactococcal bacteriophages. *Arch. Virol.* 151 1039–1053 16453083 10.1007/s00705-005-0709-4

[B19] Crutz-Le Coq A. M. Cesselin B. Commissaire J. Anba J. (2002). Sequence analysis of the lactococcal bacteriophage bIL170: insights into structural proteins and HNH endonucleases in dairy phages. *Microbiology* 148 985–1001 11932445 10.1099/00221287-148-4-985

[B20] Daly C. Fitzgerald G. F. Davis R. (1996). Biotechnology of lactic acid bacteria with special reference to bacteriophage resistance. *Antonie Van Leeuwenhoek* 70 99–110 8879402 10.1007/BF00395928

[B21] del Rio B. Binetti A. G. Martin M. C. Fernandez M. Magadan A. H. Alvarez M. A. (2007). Multiplex PCR for the detection and identification of dairy bacteriophages in milk. *Food Microbiol.* 24 75–81 16943097 10.1016/j.fm.2006.03.001

[B22] Deveau H. Labrie S. J. Chopin M. C. Moineau S. (2006). Biodiversity and classification of lactococcal phages. *Appl. Environ. Microbiol.* 72 4338–4346 16751549 10.1128/AEM.02517-05 PMC1489595

[B23] Dupont K. Vogensen F. K. Josephsen J. (2005). Detection of lactococcal 936-species bacteriophages in whey by magnetic capture hybridization PCR targeting a variable region of receptor-binding protein genes. *J. Appl. Microbiol.* 98 1001–1009 15752347 10.1111/j.1365-2672.2005.02548.x

[B24] Dupont K. Vogensen F. K. Neve H. Bresciani J. Josephsen J. (2004). Identification of the receptor-binding protein in 936-species lactococcal bacteriophages. *Appl. Environ. Microbiol.* 70 5818–5824 15466519 10.1128/AEM.70.10.5818-5824.2004 PMC522089

[B25] Durmaz E. Klaenhammer T. R. (2007). Abortive phage resistance mechanism AbiZ speeds the lysis clock to cause premature lysis of phage-infected *Lactococcus lactis*. *J. Bacteriol.* 189 1417–1425 17012400 10.1128/JB.00904-06 PMC1797342

[B26] Fortier L. C. Bouchard J. D. Moineau S. (2005). Expression and site-directed mutagenesis of the lactococcal abortive phage infection protein AbiK. *J. Bacteriol.* 187 3721–3730 15901696 10.1128/JB.187.11.3721-3730.2005 PMC1112063

[B27] Gabs S. Josephsen J. (2003). Improvement of phage defence in *Lactococcus lactis* by introduction of the plasmid encoded restriction and modification system LlaAI. *Lett. Appl. Microbiol.* 36 332–336 12680948 10.1046/j.1472-765x.2003.01320.x

[B28] Garvey P. Fitzgerald G. F. Hill C. (1995). Cloning and DNA sequence analysis of two abortive infection phage resistance determinants from the lactococcal plasmid pNP40. *Appl. Environ. Microbiol.* 61 4321–4328 8534099 10.1128/aem.61.12.4321-4328.1995 PMC167743

[B29] Guglielmotti D. M. Mercanti D. J. Reinheimer J. A Quiberoni Adel L. (2011). Review: efficiency of physical and chemical treatments on the inactivation of dairy bacteriophages. *Front. Microbiol. *2:282. 10.3389/fmicb.2011.00282 PMC3257867 22275912

[B30] Haaber J. Moineau S. Fortier L. C. Hammer K. (2008). AbiV, a novel antiphage abortive infection mechanism on the chromosome of *Lactococcus lactis* subsp. *cremoris* MG1363. *Appl. Environ. Microbiol*. 74 6528–6537 18776030 10.1128/AEM.00780-08 PMC2576692

[B31] Heap H. A. Lawrence R. C. (1976). The selection of starter strains for cheesemaking. *N. Z. J. Dairy Sci. Technol.* 11 16–20

[B32] Hejnowicz M. S. Golebiewski M. Bardowski J. (2009). Analysis of the complete genome sequence of the lactococcal bacteriophage bIBB29. *Int. J. Food Microbiol.* 131 52–61 18644641 10.1016/j.ijfoodmicro.2008.06.010

[B33] Josephsen J. Petersen A. Neve H. Nielsen E. W. (1999). Development of lytic *Lactococcus lactis* bacteriophages in a Cheddar cheese plant. *Int. J. Food Microbiol.* 50 163–171

[B34] Kleppena H. P. Bangb T. Nesa I. F. Helge Holoa B. (2011). Bacteriophages in milk fermentations: diversity fluctuations of normal and failed fermentations. *Int. Dairy J.* 21 592–600

[B35] Kong J. Josephsen J. (2002). The ability of the plasmid-encoded restriction and modification system LlaBIII to protect *Lactococcus lactis* against bacteriophages. *Lett. Appl. Microbiol.* 34 249–253 11940153 10.1046/j.1472-765x.2002.01089.x

[B36] Labrie S. Moineau S. (2000). Multiplex PCR for detection and identification of lactococcal bacteriophages. *Appl. Environ. Microbiol.* 66 987–994 10698762 10.1128/aem.66.3.987-994.2000 PMC91933

[B37] Labrie S. J. Josephsen J. Neve H. Vogensen F. K. Moineau S. (2008). Morphology, genome sequence, and structural proteome of type phage P335 from *Lactococcus lactis*. *Appl. Environ. Microbiol.* 74 4636–4644 18539805 10.1128/AEM.00118-08 PMC2519342

[B38] Lavigne R. Darius P. Summer E. J. Seto D. Mahadevan P. Nilsson A. S. Ackermann H. W. Kropinski A. M. (2009). Classification of Myoviridae bacteriophages using protein sequence similarity. *BMC Microbiol.* 9 224 10.1186/1471-2180-9-224 PMC2771037 19857251

[B39] Lillehaug D. (1997). An improved plaque assay for poor plaque-producing temperate lactococcal bacteriophages. *J. Appl. Microbiol.* 83 85–90 9246774 10.1046/j.1365-2672.1997.00193.x

[B40] Ly-Chatain M. H. Durand L. Rigobello V. Vera A. Demarigny Y. (2011). Direct quantitative detection and identification of lactococcal bacteriophages from milk and whey by real-time PCR: application for the detection of lactococcal bacteriophages in goat’s raw milk whey in France. *Int. J. Microbiol.* 2011 594369 10.1155/2011/594369 PMC3195528 22013446

[B41] Madera C. Monjardin C. Suarez J. E. (2004). Milk contamination and resistance to processing conditions determine the fate of *Lactococcus lactis* bacteriophages in dairies. *Appl. Environ. Microbiol.* 70 7365–7371 15574937 10.1128/AEM.70.12.7365-7371.2004 PMC535134

[B42] Mahony J. Deveau H. Mc Grath S. Ventura M. Canchaya C. Moineau S. Fitzgerald G. F Van Sinderen D. (2006). Sequence and comparative genomic analysis of lactococcal bacteriophages jj50, 712 and P008: evolutionary insights into the 936 phage species. *FEMS Microbiol. Lett.* 261 253–261 16907729 10.1111/j.1574-6968.2006.00372.x

[B43] Mahony J. McGrath S. Fitzgerald G. F Van Sinderen D. (2008). Identification and characterization of lactococcal-prophage-carried superinfection exclusion genes. *Appl. Environ. Microbiol.* 74 6206–6215 18723645 10.1128/AEM.01053-08 PMC2570291

[B44] McGrath S. Fitzgerald G. F Van Sinderen D. (2002). Identification and characterization of phage-resistance genes in temperate lactococcal bacteriophages. *Mol. Microbiol*. 43 509–520 11985726 10.1046/j.1365-2958.2002.02763.x

[B45] McGrath S. Seegers J. F. Fitzgerald G. F Van Sinderen D. (1999). Molecular characterization of a phage-encoded resistance system in *Lactococcus lactis*. *Appl. Environ. Microbiol.* 65 1891–1899 10223975 10.1128/aem.65.5.1891-1899.1999 PMC91272

[B46] McIntyre K. Heap H. A. Davey G. P Limsowtin G. K. Y. (1991). The distribution of lactococcal bacteriophage in the environment of a cheese manufacturing plant. *Int. Dairy J.* 1 183–197

[B47] Michelsen O. Cuesta-Dominguez A. Albrechtsen B. Jensen P. R. (2007). Detection of bacteriophage-infected cells of *Lactococcus lactis* by using flow cytometry. *Appl. Environ. Microbiol.* 73 7575–7581 17921265 10.1128/AEM.01219-07 PMC2168076

[B48] Miklič A. Rogelj I. (2003). Characterization of lactococcal bacteriophages isolated from Slovenian dairies. *Int. J. Food Sci. Technol.* 38 305–311

[B49] Mills S. Griffin C. Coffey A. Meijer W. C. Hafkamp B. Ross R. P. (2010). CRISPR analysis of bacteriophage-insensitive mutants (BIMs) of industrial *Streptococcus thermophilus*-implications for starter design. *J. Appl. Microbiol.* 108 945–955 19709335 10.1111/j.1365-2672.2009.04486.x

[B50] Moineau S. Borkaev M. Holler B. J. Walker S. A. Kondo J. K. Vedamuthu E. R. Vandenbergh P. A. (1996). Isolation and characterization of lactococcal bacteriophages from cultured buttermilk plants in the United States. *J. Dairy Sci.* 79 2104–2111

[B51] Moineau S. Fortier J. Ackermann H. W. Pandian S. (1992). Characterization of lactococcal bacteriophages from Quebec cheese plants. *Can. J. Microbiol.* 38 875–882

[B52] Moisan M. Moineau S. (2012). Multilocus sequence typing scheme for the characterization of 936-like phages infecting *Lactococcus lactis*. *Appl. Environ. Microbiol.* 78 4646–4653 22522686 10.1128/AEM.00931-12 PMC3370485

[B53] Muller-Merbach M. Neve H. Hinrichs J. (2005a). Kinetics of the thermal inactivation of the *Lactococcus lactis* bacteriophage P008. *J. Dairy Res.* 72 281–286 16174358 10.1017/S0022029905000725

[B54] Muller-Merbach M. Rauscher T. Hinrichs J. (2005b). Inactivation of bacteriophages by thermal and high-pressure treatment. *Int. Dairy J.* 15 777–784

[B55] Neve H. Berger A. Heller K. J. (1995). A method for detecting and enumerating airborne virulent bacteriophage of dairy starter cultures. *Kieler Milchw. Forsch.* 47 193–207

[B56] Neve H. Kemper U. Geis A. Heller K. J. (1994). Monitoring and characterization of lactococcal bacteriophages in a dairy plant. *Kieler Milchw. Forsch.* 46 167–178

[B57] Neve H. Laborius A. Heller K. J. (2003). Testing of the applicability of battery-powered portable microbial air samplers for detection and enumeration of air- borne *Lactococcus lactis* dairy bacteriophages. *Kieler Milchw. Forsch.* 55 301–315

[B58] O’Driscoll J. Glynn F. Cahalane O. O’Connell-Motherway M. Fitzgerald G. F Van Sinderen D. (2004). Lactococcal plasmid pNP40 encodes a novel, temperature-sensitive restriction-modification system. *Appl. Environ. Microbiol.* 70 5546–5556 15345443 10.1128/AEM.70.9.5546-5556.2004 PMC520859

[B59] Parada J. L Fabrizio S. V. D. (2001). Estabilidad de fagos de *Lactococcus lactis* frente al hipoclorito de sodio y durante el almacenamiento. *Rev. Argent. Microbiol.* 33 89–95 11494761

[B60] Quiberoni A. Guglielmotti D. M. Reinheimer J. A. (2003). Inactivation of *Lactobacillus delbrueckii* bacteriophages by heat and biocides. *Int. J. Food Microbiol.* 84 51–62 12781954 10.1016/s0168-1605(02)00394-x

[B61] Quiberoni A. Suarez V. B. Reinheimer J. A. (1999). Inactivation of Lactobacillus helveticus bacteriophagesby thermal and chemical treatments. *J. Food Prot.* 62 894–898 10456743 10.4315/0362-028x-62.8.894

[B62] Raiski A. Belyasova N. (2009). Biodiversity of *Lactococcus lactis* bacteriophages in the Republic of Belarus. *Int. J. Food Microbiol.* 130 1–5 19195733 10.1016/j.ijfoodmicro.2008.12.024

[B63] Ricagno S. Campanacci V. Blangy S. Spinelli S. Tremblay D. Moineau S. Tegoni M. Cambillau C. (2006). Crystal structure of the receptor-binding protein head domain from *Lactococcus lactis* phage bIL170. *J. Virol.* 80 9331–9335 16940545 10.1128/JVI.01160-06 PMC1563906

[B64] Rousseau G. M. Moineau S. (2009a). Evolution of *Lactococcus lactis* phages within a cheese factory. *Appl. Environ. Microbiol.* 75 5336–5344 19542338 10.1128/AEM.00761-09 PMC2725462

[B65] Rousseau G. V. M. Moineau S. (2009b). Evolution of *Lactococcus lactis* phages within a cheese factory. *Appl. Environ. Microbiol.* 75 5336–5344 19542338 10.1128/AEM.00761-09 PMC2725462

[B66] Sciara G. Bebeacua C. Bron P. Tremblay D. Ortiz-Lombardia M. Lichiere J. Van Heel M. Campanacci V. Moineau S. Cambillau C. (2010). Structure of lactococcal phage p2 baseplate and its mechanism of activation. *Proc. Natl. Acad. Sci. U.S.A.* 107 6852–6857 20351260 10.1073/pnas.1000232107 PMC2872406

[B67] Siponen M. Sciara G. Villion M. Spinelli S. Lichiere J. Cambillau C. Moineau S. Campanacci V. (2009a). Crystal structure of ORF12 from *Lactococcus lactis* phage p2 identifies a tape measure protein chaperone. *J. Bacteriol.* 191 728–734 19047351 10.1128/JB.01363-08 PMC2632072

[B68] Siponen M. Spinelli S. Blangy S. Moineau S. Cambillau C. Campanacci V. (2009b). Crystal structure of a chimeric receptor binding protein constructed from two lactococcal phages. *J. Bacteriol.* 191 3220–3225 19286807 10.1128/JB.01637-08 PMC2687176

[B69] Soukoulis C. Panagiotidis P. Koureli R. Tzia C. (2007). Industrial yogurt manufacture: monitoring of fermentation process and improvement of final product quality. *J. Dairy Sci.* 90 2641–2654 17517704 10.3168/jds.2006-802

[B70] Spinelli S. Desmyter A. Verrips C. T. De Haard H. J. Moineau S. Cambillau C. (2006). Lactococcal bacteriophage p2 receptor-binding protein structure suggests a common ancestor gene with bacterial and mammalian viruses. *Nat. Struct. Mol. Biol.* 13 85–89 16327804 10.1038/nsmb1029

[B71] Suarez V. Moineau S. Reinheimer J. Quiberoni A. (2008). Argentinean *Lactococcus lactis* bacteriophages: genetic characterization and adsorption studies. *J. Appl. Microbiol.* 104 371–379 17887981 10.1111/j.1365-2672.2007.03556.x

[B72] Suarez V. B. Reinheimer J. A. (2002). Effectiveness of thermal treatments and biocides in the inactivation of Argentinian *Lactococcus lactis* phages. *J. Food Prot.* 65 1756–1759 12430698 10.4315/0362-028x-65.11.1756

[B73] Sun X. Gohler A. Heller K. J. Neve H. (2006). The ltp gene of temperate *Streptococcus thermophilus* phage TP-J34 confers superinfection exclusion to *Streptococcus thermophilus* and *Lactococcus lactis*. *Virology* 350 146–157 16643978 10.1016/j.virol.2006.03.001

[B74] Svensson U. Christiansso A. (1991). Methods for phage monitoring. *Bull. Int. Dairy Fed.* 29–39

[B75] Szczepanska A. K. Hejnowicz M. S. Kolakowski P. Bardowski J. (2007). Biodiversity of *Lactococcus lactis* bacteriophages in Polish dairy environment. *Acta Biochim. Pol.* 54 151–158 17311108

[B76] Tremblay D. M. Tegoni M. Spinelli S. Campanacci V. Blangy S. Huyghe C. Desmyter A. Labrie S. Moineau S. Cambillau C. (2006). Receptor-binding protein of *Lactococcus lactis* phages: identification and characterization of the saccharide receptor-binding site. *J. Bacteriol.* 188 2400–2410 16547026 10.1128/JB.188.7.2400-2410.2006 PMC1428394

[B77] Twomey D. P. De Urraza P. J. Mckay L. L O’Sullivan D. J. (2000). Characterization of AbiR, a novel multicomponent abortive infection mechanism encoded by plasmid pKR223 of *Lactococcus lactis* subsp. *lactis* KR2. *Appl. Environ. Microbiol.* 66 2647–2651 10831451 10.1128/aem.66.6.2647-2651.2000 PMC110594

[B78] Verreault D. Gendron L. Rousseau G. M. Veillette M. Masse D. Lindsley W. G. Moineau S. Duchaine C. (2011). Detection of airborne lactococcal bacteriophages in cheese manufacturing plants. *Appl. Environ. Microbiol.* 77 491–497 21115712 10.1128/AEM.01391-10 PMC3020544

